# *SMAD4* Somatic Mutations in Head and Neck Carcinoma Are Associated With Tumor Progression

**DOI:** 10.3389/fonc.2019.01379

**Published:** 2019-12-06

**Authors:** Li-Han Lin, Kuo-Wei Chang, Hui-Wen Cheng, Chung-Ji Liu

**Affiliations:** ^1^Department of Medical Research, MacKay Memorial Hospital, Taipei, Taiwan; ^2^Department of Stomatology, Taipei Veterans General Hospital, Taipei, Taiwan; ^3^School of Dentistry, Institute of Oral Biology, National Yang-Ming University, Taipei, Taiwan; ^4^Department of Oral and Maxillofacial Surgery, Taipei MacKay Memorial Hospital, Taipei, Taiwan

**Keywords:** head and neck cancer, loss of heterozygosity, *SMAD4*, somatic mutation, survival

## Abstract

As the incidence and the mortality rate of head and neck squamous cell carcinoma (HNSCC) is increasing worldwide, gaining knowledge about the genomic changes which happen in the carcinogenesis of HNSCC is essential for the diagnosis and therapy of the disease. *SMAD4* (DPC4) is a tumor suppressor gene. It is located at chromosome 18q21.1 and a member of the SMAD family. Which mediates the TGF-β signaling pathway, thereby controlling the growth of epithelial cells. In the study presented here, we analyzed tumor samples by multiplex PCR-based next-generation sequencing (NGS) and found deleterious mutations of *SMAD4 in* 4.1% of the tumors. Knock-down experiments of endogenous and exogenous SMAD4 expression demonstrated that SMAD4 is involved in the migration and invasion of HNSCC cells. Functional analysis of a missense mutation in the MH1 domain of SMAD4 may be responsible for the loss of function in suppressing tumor progression. Missense *SMAD4* mutations, therefore, could be useful prognostic determinants for patients affected by HNSCCs. This report is the first study where NGS analysis based on multiplex-PCR is used to demonstrate the imminent occurrence of missense *SMAD4* mutations in HNSCC cells. The gene analysis that we performed may support the identification of *SMAD4* mutations as a diagnostic marker or even as a potential therapeutic target in head and neck cancer. Moreover, the analytic strategy proposed for the detection of mutations in the *SMAD4* gene may be validated as a platform to assist mutation screening.

## Introduction

Head and neck squamous cell carcinomas (HNSCC), which include oral squamous cell carcinomas, are the sixth most prevalent malignancy worldwide (1, 2). The pathogenesis of HNSCC is affected by many molecular factors, among them e.g., mutations in *TP53*, and genes related to *PIK3CA and* Notch family signaling (3–9). The role which these factors are playing in the progression of the tumors remains unclear to a wide extent (3, 4, 6, 10, 11). Although the knowledge on head and neck carcinogenesis has improved a lot in the past 40 years and many innovations in surgery as well as chemo- and radiotherapy have been made, the survival rates for many HNSCC types have not improved considerably (2). Recent developments in high-throughput, next-generation parallel sequencing technologies are facilitating sensitive detection, and quantification of genetic alterations. New insights in the molecular basis of HNSCC progression have been provided by whole-exome sequencing (WES) (8, 12, 13). The analysis of WES data which was obtained from The Cancer Genome Atlas (TCGA) (6, 14) pointed to novel genes with significant mutations and underlined the complex molecular pathogenesis of HNSCC, which includes a high degree of heterogeneity between tumors (15).

SMAD4 is a transcription factor of the SMAD family which takes part in TGF-β signaling. SMAD4 stands for SMA- and MAD-related protein 4, other names are SMAD family member 4, Deleted in Pancreatic Cancer-4 (DPC4) and Mothers against decapentaplegic homolog 4, SMAD4 is present in all metazoans and is highly conserved between species (16). As the main effector of TGF-β signaling, SMAD4 has been found to be non-functional in more than half of adenocarcinomas of the pancreatic duct (17–19), and to varying degrees, in several other types of cancers (20–24). In many studies that have been conducted in the past 20 years it was found that the loss of SMAD4 function alone does not initiate a tumor, but it may promote the progression of tumors which have been initiated by other molecular defects, like the activation of KRas activation in the case of pancreatic duct adenocarcinoma and the inactivation of APC in colorectal cancer (20, 24). The loss of SMAD4 is playing a crucial role in the response to DNA damage with the consequence of increased genomic instability. This is very prominent in skin cancer and suggests a distinct role of SMAD4 in the progression of various types of tumors (25).

Screening large genes with multiples exons for mutations by traditional Sanger sequencing is slow, labor intensive and costly. Next-generation sequencing (NGS) in contrast allows the direct analysis of mutations in monogenic diseases at low cost without pre-screening. Novel DNA variants that have been identified by NGS still may be corroborated by Sanger sequencing before reporting them.

In this study, we performed NGS analysis based on multiplex PCR NGS for the investigation of *SMAD4* mutations in HNSCC.

We found that mutations of *SMAD4*, as well as its expression level, are linked to the progression of HNSCC and affect patient survival. Moreover, we investigated the role of deleterious *SMAD4* mutations to elucidate their role in HNSCC neoplasms.

## Materials and Methods

### Patients

This study was approved by the Institutional Review Board of Mackay Memorial Hospital (approval number: 15MMHIS104). All patients provided written informed consent. Tumor samples were obtained from 122 patients undergoing HNSCC surgery (Supplementary Table 1). Cells were isolated from tissue sections by laser capture microdissection (LCM) following established protocols (3, 4). Additionally, 10 mL of whole blood was collected in the morning after fasting in Vacutainer tubes containing EDTA as the anticoagulant (Becton Dickson, Franklin Lakes, NJ, USA) from each patient. None of the patients included in this study had received radiotherapy or adjuvant chemotherapy before surgery. DNA was extracted from blood and cancerous tissue as reported previously (26).

### *SMAD4* Mutation Analysis by PCR-Based NGS

Individual primer sets for 10 long PCR reactions were designed with Primer3 (version 0.4.0) (ELIXIR, funded by the European Commission) to amplify the entire coding sequence (exons 2–12) for human *SMAD4* (Supplementary Table 2 and Supplementary Figure 1A). Amplicon concentrations were determined with the Qubit dsDNA HS Assay kit on a Qubit 2.0 fluorometer (Life Technologies, Carlsbad, CA, USA). PCR reactions were carried out using the KAPA LongRange HotStart kit (Kapa Biosystems, Wilmington, MA, USA). For library generation, long PCR products of each sample were pooled and then purified using Agencourt Ampure XP beads (Beckman Coulter, Pasadena, CA, USA). Indexed libraries of the pooled PCR products were prepared using the Illumina Nextera XT library preparation kit and then sequenced on the Illumina MiSeq system, following the manufacturer's instructions. Variant call format files were generated using the MiSeq Reporter software (version 2.3.32). The variants were further filtered on the basis of the following criteria: (1) DP <30, (2) genotype quality <30, (3) number of mismatches within a 40-bp window ≤3, (4) mutant allele frequency of at least 10% in tumors and <1% in normal cells, and (5) MAF in the 1000 Genomes Project and dbSNP137 > 1%. The Integrative Genomics Viewer (IGV) was used to determine the read counts of the target amplicons and to confirm the detected variants. The mutation spectrum and lollipop figures for *SMAD4* were generated with the OncoPrinter and MutationMapper tools available at cBioPortal (27, 28).

### Mutation Point Validation

To validate the somatic mutations which were identified in the multiplex PCR-based NGS results, conventional Sanger sequencing was applied. Individual primer sets were designed in Primer3 (version 0.4.0), they have been listed in Supplementary Table 3. For Sanger sequencing, PCR reactions were performed with a standard hot start kit. Amplicons were sequenced with the ABI BigDye Terminator Cycle Sequencing kit on an ABI 3730xl DNA analyzer (Applied Biosystems, Foster City, CA, USA).

### Immunohistochemistry

SMAD4 protein was visualized in tissue sections by immunohistochemistry, following previously reported protocols (29). In brief, 5-μm-thick tissue sections were dewaxed, rehydrated, and then incubated with monoclonal mouse anti-human SMAD4 antibody (sc-7966, 1:100 dilution; Santa Cruz Biotechnology, Santa Cruz, CA, USA) in a humidification chamber at 4°C overnight. After rinsing with PBS, standard immunohistochemical staining was done using streptavidin-biotin complex system (Dako Corp.) with aminoethylcarbazole as the chromogen and subsequently counterstained with hematoxylin and mounted with Clearmount (Zymed Laboratories, Inc.). The primary antibody used was a Preimmune mouse IgG was used as a negative control. Normal epithelium adjacent to the tumor served as the internal positive control. Tumors containing ≥50% and <50% positive cancer cells were classified to have high and low SMAD4 expression (30).

### Cell Culture, Reagents, and Phenotypic Assays

In this study, the HNSCC cell lines SAS, OECM-1, HSC3, FaDu, SCC25, OC3, and OC4 were used. Normal human oral keratinocytes (NOKs); served as controls. The cells were cultured as described previously (31). Our cell lines were authenticated by Mission Biotech (Nangang, Taipei, Taiwan) on August 8, 2017, using the Promega StemElite ID System and analyzed on ABI PRISM 3730 Genetic Analyzer with GeneMapper (version 3.7). si-*SMAD4* and scramble control (si-control) oligonucleotides were purchased from Santa Cruz Biotech (Santa Cruz). For transfection, TransFectin Lipid Reagent (BioRad Lab., Hercules, CA, USA) was used. sh-SMAD4 vectors (TRCN0000010321) and a sh-Luc control vector (TRCN0000072249), packaged in lentiviruses, were purchased from National RNAi Core (Academia Sinica, Taipei, Taiwan). The cells were infected and selected using puromycin (Sigma-Aldrich) at 5.0 μg/mL for 7 days to establish stable subclones. Phenotypic events, including proliferation, migration, and invasion, were analyzed as previously described (10, 32).

### Analysis of SMAD4 Mutations in HNSCC Cell Lines

All coding exons of *SMAD4* in SAS, OECM-1, HSC3, FaDu, SCC25, OC3, and OC4 cells were amplified by PCR and then sequenced using the ABI BigDye Terminator Cycle Sequencing kit on an ABI 3730xl DNA analyzer (Applied Biosystems). The variants with MAF > 1% in the 1000 Genomes Project and dbSNP137 were filtered.

### Constructs

The *SMAD4* cDNA sequence was amplified from the cDNA of SAS cells with the primers *SMAD4*_Forward and *SMAD4*_Reverse, introducing BamHI and EcoRI sites for directional cloning. The PCR product then was subcloned into the pBabe puro vector. The p.H132Y, p.P296T, and p.A488V mutations were introduced into *SMAD4* cDNA with the Q5 Site-Directed Mutagenesis kit (New England BioLabs, Ipswich, MA, USA) with the primers H132Ymut, P296Tmut, and A488Vmut. We named the resulting mutants p.H132Y, p.P296T, and p.A488V, respectively. Supplementary Table 3 lists the primers used in this section.

### Loss of Heterozygosity Analysis at the *SMAD4* Locus

Three polymorphic markers close to the *SMAD4* locus (D18S363, D18S474, and D18S46) were used to analyze the loss of heterozygosity status (LOH) of *SMAD4*. These markers are described in Supplementary Table 3. As templates, samples of genomic DNA (~100 ng) were extracted from HNSCCs and matching normal tissues. The PCR reaction (20 μL) contained 5 × Phusion HF buffer, 200 μM of each dNTP, 0.25 μM of each marker, and 0.3 μl (0.6 units) of Phusion High-Fidelity DNA Polymerase (Thermo Fisher Scientific, Vilnius, Lithuania). Amplification was performed under the following conditions: 98°C for 5 min, followed by 35 cycles at 98°C for 20 s, 60°C for 15 s, and 72°C for 30 s. The final extension was at 72°C for 7 min. The PCR product (0.5 μL) was mixed with 0.5 μL of GeneScan-600 LIZ dye Size Standard (Applied Biosystems) in 10 μL of Hi-Di formamide (Applied Biosystems), denatured for 3 min at 95°C, and then cooled on ice. The samples were separated by capillary electrophoresis with an ABI PRISM 96-capillary 3730xl DNA Analyzer (Applied Biosystems) and the results were analyzed using GeneMapper (version 3.7; Applied Biosystems). LOH was evaluated using the following formula: LOH = (height of tumor allele 2/height of tumor allele 1)/(height of normal allele 2/height of normal allele 1). As described previously (33). When the height of the tumor alleles decreased by >40%, the calculated LOH became >1.49 or <0.5; thus, we considered this ratio to indicate LOH positivity (Supplementary Figure 1B). Homozygous cases were considered non-informative for LOH.

### Western Blotting

Western Blot analysis was performed as previously described (29). Equal amounts of protein (30 μg) were loaded per lane. As primary antibodies anti-SMAD4 (diluted 1:500, Santa Cruz) and anti-GAPDH (diluted 1:5,000, Santa Cruz) antibodies were employed. For detection, an HRP-conjugated horse anti-mouse IgG was used as the secondary antibody (diluted 1:5,000; Cell signaling; Cell Signaling Technology, Danvers, MA, USA).

### Statistical Analysis

Data are presented as the mean ± SEM. Chi-square and *t*-tests were used. Overall survival (OS) was defined as the time between the date of first diagnosis and the date of death or final follow-up. Disease-free survival (DFS) was defined as the time from the date of first diagnosis until the date of first recurrence or death. Patients without evidence of disease recurrence were censored at the final follow-up or death. Kaplan–Meier analysis was used to compare OS and DFS between the two groups. The multivariate Cox Proportional Hazards Model was used to assess the association of both OS and DFS with *SMAD4* LOH, mutation status, and immunoexpression. Statistical significance was assumed to be indicated by ^*^P < 0.05, ^**^P < 0.01, and ^***^P < 0.001 respectively. Cross-comparisons showing no statistically significant differences were not considered in further analysis.

## Results

### Somatic Mutations and LOH in *SMAD4*

**Figure 1:**
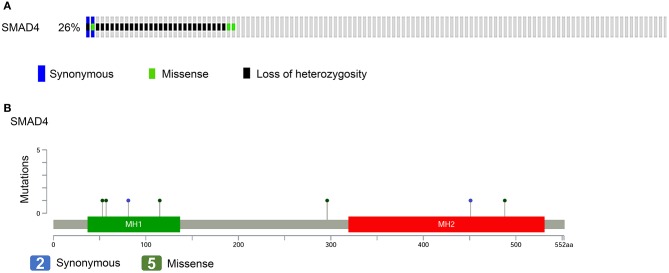
*SMAD4* mutations in patients with HNSCC. **(A)** Frequency of *SMAD4* LOH, mutations, or both. Each column represents one patient. Colored boxes designate LOH status and mutation types. **(B)** Distribution of mutations in the sequence coding for *SMAD4*. Colored ovals represent mutation types, colored boxes represent domains. Colored boxes represent mutation types.

For the evaluation of the performance of SMAD4 in clinical assessments, 122 HNSCC samples were analyzed by multiplex PCR-based NGS and LOH analysis. We identified seven somatic mutations in the samples by NGS; of these, two were synonymous and five were missense mutations (Figures 1A,B, Supplementary Table 4). No hotspot region was found for *SMAD4* mutations. The missense mutation p.Ala488Val, previously has been reported by Lee et al. (chr18:48604641) (34). Three polymorphic biomarkers (D18S474, D18S46, and D18S363) which are surrounding the *SMAD4* locus were used to analyze LOH status. Percent LOH was 15.57% (19/122), 13.93% (17/122), and 13.11% (16/122) in D18S363, D18S46, and D18S474, respectively. In 28 (22.95%) patients, LOH was detected in at least one of the three markers (Figure 1A), whereas LOH was noted in two or all three markers in 17 (13.93%) patients.

**Figure 2:**
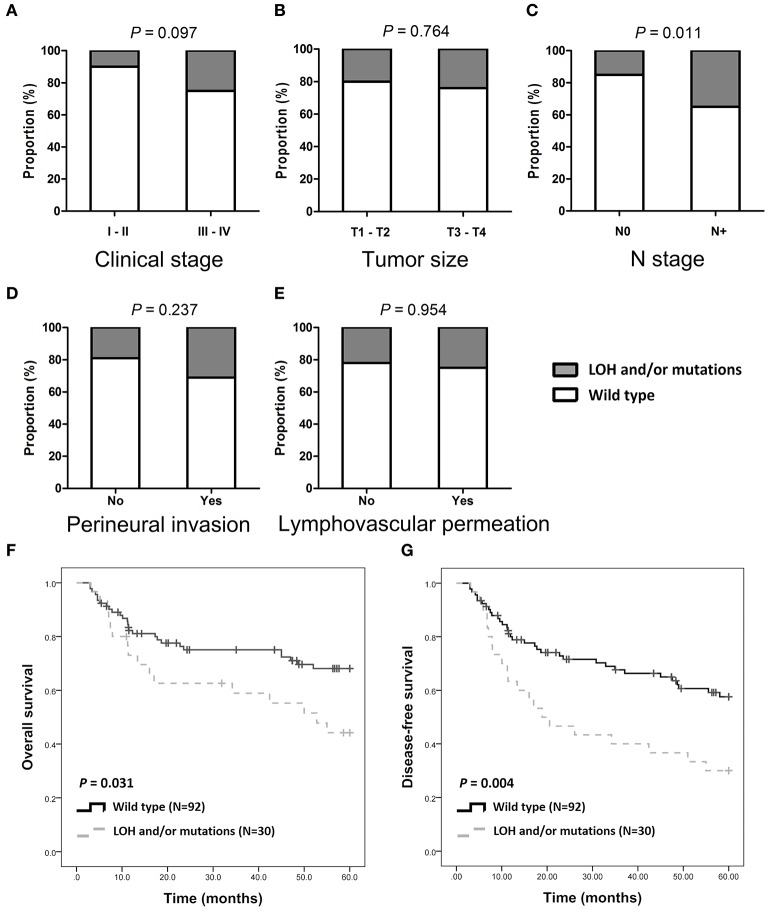
*SMAD4* LOH and mutations and clinical/pathological parameters. **(A–E)** Histograms are showing the association of *SMAD4* LOH with mutation status and clinical stages **(A)**, tumor size **(B)**, N stage **(C)**, perineural invasion **(D)**, and lymphovascular permeation **(E)**. **(F,G)** Kaplan–Meier analysis according to *SMAD4* LOH and mutation status for OS **(F)** and DFS **(G)**.

Of the 122 patients, 32 (26.2%) had SMAD4 LOH, mutations, or both, and the remaining 90 demonstrated no mutations or LOH (Figure 1A). The frequency of *SMAD4* LOH was higher in patients with lymph node metastasis, mutations, or both compared to those where no metastasis was observed (*P* = 0.011, Figure 2C). No significant differences were observed in the clinical stage, tumor size, perineural invasion, or lymphovascular permeation (Figures 2A–E, Supplementary Table 5). The Kaplan–Meier analysis indicated that patients with *SMAD4* LOH, mutations, or both had a significantly poorer OS and DFS than those with wild-type *SMAD4* (*P* = 0.031 and 0.004, respectively; Figures 2F,G, respectively). Both univariate and adjusted multivariate Cox regression analyses revealed poor OS and DFS in patients with *SMAD4* LOH, mutations, or both (OS hazard ratio [HR]: 1.95, *P* = 0.034; OS adjusted HR: 2.08, *P* = 0.024; DFS HR: 2.08, *P* = 0.008, DFS adjusted HR: 1.97, *P* = 0.016; Table 1).

**Table 1:** Univariate and Multivariate analysis of disease-free survival rate in HNSCC patients.

**Variables**	**HR (95%CI)**	***P***	**Adjusted HR (95%CI)**	***P***
Overall survival				
*SMAD4* LOH and mutations				
Wild type	Reference		Reference	
LOH and/or mutations	1.95 (1.05–3.62)	0.034^*^	2.08 (1.10–3.92)	0.024^*^
*SMAD4* expression				
High	Reference		Reference	
Low	4.30 (1.02–18.15)	0.047^*^	4.09 (0.93–18.00)	0.063
Disease-free survival				
*SMAD4* LOH and mutations				
Wild type	Reference		Reference	
LOH and/or mutations	2.08 (1.21–3.57)	0.008^*^	1.97 (1.14–3.42)	0.016^*^
*SMAD4* expression				
High	Reference		Reference	
Low	4.43 (1.05–18.69)	0.043^*^	4.54 (1.03–20.12)	0.046^*^

*Adjusted for age, gender, and clinical stage*.

*HR, hazard ratio; CI, confidence interval*.

### SMAD4 Expression in HNSCC Patients

**Figure 3:**
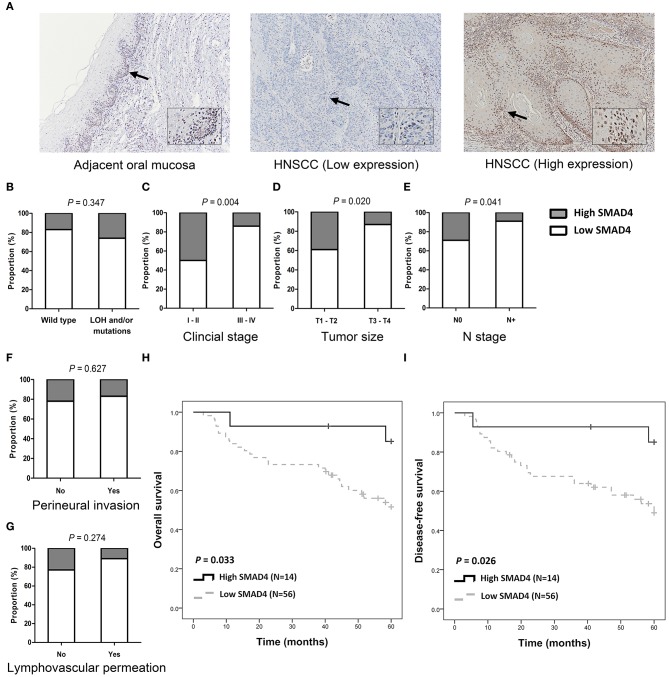
SMAD4 immunoexpression and clinicopathological parameters. **(A)** Immunohistochemistry for SMAD4 in adjacent normal-appearing mucosa (left) and HNSCC tumor tissue (middle and right), as indicated. Tumor tissue showing low (middle), and high (right) SMAD4 staining. Arrows indicate enlarged area. Inset images enlarged by 300% relative to original image. **(B–G)** Histograms showing the association of SMAD4 immunoexpression with *SMAD4* LOH and mutation status **(B)**, clinical stage **(C)**, tumor size **(D)**, N stage **(E)**, perineural invasion **(F)**, and lymphovascular permeation **(G)**. **(H,I)** Kaplan–Meier analysis according to SMAD4 immunoexpression for OS **(H)** and DFS **(I)**.

SMAD4 was detected in the cytoplasm and in the nuclei of basal and parabasal cells in normal oral epithelium which was adjacent to tumors (Figure 3A, top left). In tumor tissues, SMAD4 immunoreactivity varied from weak (Figure 3A, middle) to intense (Figure 3A, right). Low SMAD4 expression significantly correlated with the clinical stage (*P* = 0.004), tumor size (*P* = 0.020), and lymph node metastasis (*P* = 0.041; Figures 3B–G and Supplementary Table 6). No differences were observed in SMAD4 expression in association with the LOH and mutation status, perineural invasion, and lymphovascular permeation. The Kaplan–Meier analysis revealed significantly poorer OS and DFS in patients when SMAD4 expression was low, compared to those with high SMAD4 expression (*P* = 0.033 and *P* = 0.026, respectively; Figures 3H,I, respectively). DFS was shorter in patients with when SMAD4 expression was low, as revealed by both univariate and adjusted multivariate Cox regression analysis. (HR: 4.43, *P* = 0.043 and HR: 4.54, *P* = 0.046, respectively; Table 1). Univariate Cox regression analysis showed that OS was poorer in patients with low SMAD4 expression (HR: 4.30, *P* = 0.047). However, after the multivariate Cox regression analysis, the correlation between SMAD4 expression and OS became only slightly significant (HR: 4.09, *P* = 0.063).

### Association Between SMAD4 Knockdown and Increased HNSCC Invasiveness

**Figure 4:**
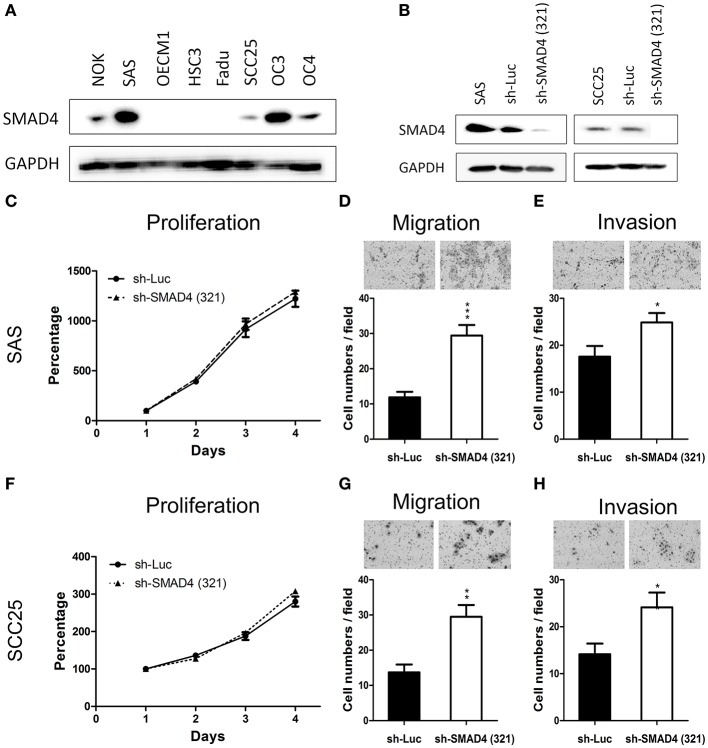
Association of *SMAD4* knockdown with oncogenic phenotypes in HNSCC cells. **(A)** Western blotting for SMAD4 expression in NOK cells and the seven HNSCC cell lines investigated. In SAS and SCC25 cells, *SMAD4* expression levels are higher compared to other cell lines. **(B)** Western blotting on extracts from sh-Luc cell subclone (control) and sh-SMA1D4 (321) cell subclone, which was established from the construct TRCN0000010321. **(C–E)** SAS and **(F–H)** SCC25 cells: proliferation (**C,F**, respectively), migration (**D,G**, respectively), and invasion (**E,H**, respectively).

The protein levels of SMAD4 in NOKs and HNSCC cells were determined by Western Blotting. As shown in Figure 4A, four of the seven (57.1%) tested HNSCC cell lines had decreased or no protein expression of SMAD4, compared to NOKs. Interestingly, in SAS and OC3 cells the endogenous SMAD4 expression relative to the NOKs was elevated. No SMAD4 protein could be detected in FaDu cells (Figure 4A). These findings are in agreement with the already described homozygous deletion of *SMAD4* in FaDu cells (35). The remaining two HNSCC cell lines (OC3 and HSC3 cells) were sequenced and two missense mutations were found (Supplementary Table 7) No mutation or LOH was found in OC4, OECM1, SAS, or SCC25 cells. OC3 and HSC3 which have missense mutation were excluded from further phenotype studies.

To further elucidate the oncogenic role of *SMAD4* in HNSCC, we established stable *SMAD4*-knockdown SAS and SCC25 subclones. The knockdown effect was confirmed through Western blotting (Figure 4B). Cell proliferation was not affected by the knockdown of *SMAD4* (Figures 4C,F), but HNSCC cell migration and invasion were significantly increased (Figures 4D,E,G,H). To corroborate this result, we transfected SAS and SCC25 cells with si-*SMAD4* oligonucleotides (Supplementary Figure 2). Western blotting confirmed the si-*SMAD4* knockdown effect on *SMAD4* expression (Supplementary Figure 2A). Migration and invasion of HNSCC cells were significantly increased during the transient knockdown, but their proliferation behavior did not change (Supplementary Figures 2B–D). These results indicate that the mobility and invasiveness of HNSCC cells were enhanced by the knockdown of *SMAD4*.

### Association Between Mutations of *SMAD4* With Increased Invasiveness in HNSCC

**Figure 5:**
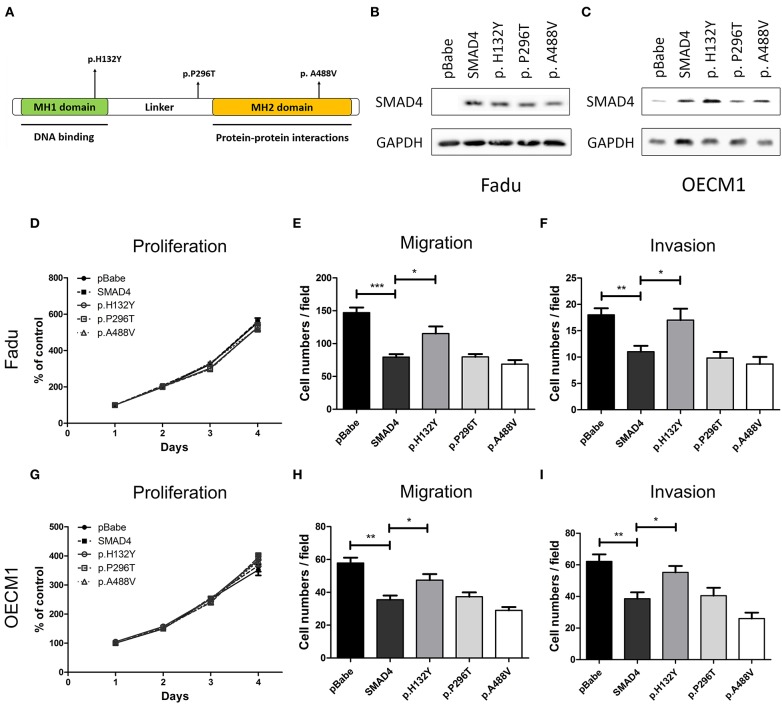
Association of SMAD4 expression with oncogenic phenotypes in HNSCC cells. **(A)** Illustration of various *SMAD4* constructs. Vertical arrows indicate the position of various mutations that were generated for this study. **(B,D–F)** FaDu and **(C,G–I)** OECM1 cells. Results of the Western blotting analysis. The cells were transfected with the vectors, and Western blotting was performed using an anti-SMAD4 antibody to detect the expression of exogenous SMAD4 proteins (**B,C**, respectively): Proliferation (**D,G**, respectively), migration (**E,H**, respectively), and invasion (**F,I**, respectively).

To characterize the effects of *SMAD4* on the phenotype, the full-length coding region of *SMAD4* was amplified from SAS cells and cloned to generate the *SMAD4* construct. In the tumor samples, three missense mutations were found (p.His132Tyr, p.Pro296Thr, and p.Ala488Val, Supplementary Table 4). These mutations are localized in the MH1, Linker, and MH2 domains, respectively (Figure 5A). By Sanger sequencing, we confirmed them as somatic mutations (Supplementary Figure 1C). We further made constructs for these mutations to be able to express these mutant proteins for further study. FaDu and OECM1 cells (which have no or only low*SMAD4* expression) were transfected with the *SMAD4* vector and the mutant constructs. Western blotting was used to detect *SMAD4* expression levels (Figures 5B,C). The exogenous *SMAD4* expression reduced the migration and invasion of FaDu and OECM1 cells (Figures 5E,F,H,I). When the mutant p.H132Y was transfected, the phenotypes repressed by *SMAD4* overexpression was abolished. The proliferation rates of the transfected cell lines were not affected (Figures 5D,G). However, transfection with p.P296T and p.A488V mutant constructs did not abolish the rescuing effects of *SMAD4* overexpression on invasion and migration in HNSCC cells.

## Discussion

Varying rates of *SMAD4* mutations have been detected in a wide range of cancers by large-scale exome sequencing. Compared with 35% of pancreatic cancer and 12% of colon cancer cases (20, 36–38), *SMAD4* mutation in other types of cancers has occurred at lower rates. In COSMIC cohort studies, point mutations of *SMAD4* were identified in 0.21, 2.24, 2.46, and 8.86% of kidney, lung, esophagus, and biliary tract cancers, respectively (24, 38–44). In general, 2.5 to 4% of the HNSCC tumors demonstrate the somatic mutation of *SMAD4*, making *SMAD4* the fourth mutated gene in different types of cancers (8). In the study presented here, somatic *SMAD4* mutations were found in 4.1% of HNSCC tumors we analyzed. This study is the first where somatic *SMAD4* mutations in HNSCC were detected by multiplex PCR-based NGS. Besides, IGV was used to reconfirm all mutations. Sanger sequencing confirmed three specific non-sense mutations. Thus, despite *SMAD4* is a large gene that is coding for a 552-amino acid polypeptide with a molecular weight of 60.439 Da, the current NGS-based strategy can be a reliable method for *SMAD4* screening. No hotspot for mutations in the *SMAD4* gene has been reported previously and we have not detected any was it observed in our study (8); therefore, allele-specific approaches that are targeting only common mutations (45, 46) are not suitable for the exploration of *SMAD4* mutations in HNSCC. We have designed the multiplex PCR assay to generate *SMAD4* amplicon libraries for sequencing. This includes protein-coding regions as well as conserved splice sites. Because this approach is highly scalable, it may provide advantages over Sanger sequencing regarding its potential application in routine clinical diagnostics, also because the labor required for analyzing individual samples for somatic *SMAD4* mutations is low.

*SMAD* proteins have two evolutionarily conserved regions separated by a linker region, MAD homology 1 and 2 (MH1 and MH2, respectively). The MH1 domain at the N/terminus is responsible for sequence-specific DNA binding (47, 48), the roles of the MH2 domain are heteromerization and transactivation (49, 50). Besides, the MH2 region partially interferes with the DNA-binding function of the MH1 region (47, 50, 51). Mutations in the domain between L43 and R135 may reduce the ability of *SMAD4* to bind DNA considerably, as a β-hairpin protein motif within this region is responsible for the interaction with DNA. Our results demonstrate that the whole MH1 domain is very sensitive to changes in its overall primary structure and that tumorigenic mutations within the area of L43 and R135 interfere with its capability to bind DNA (52). Kim et al. were the first to document a non-sense mutation of *SMAD4* (GAA526TAA) in two cell lines derived from the same HNSCC patient (53). Reiss et al. reported a homozygous deletion which includes the *SMAD4* gene locus in FaDu cells (54). Others have furthermore identified the *SMAD4* mutation in this HNSCC cell line (35, 55). The results of our Western Blotting experiments verified that this mutation which results in a nonsense mutation causes the complete loss of *SMAD4* expression. This finding is consistent with previous observations that the majority of missense mutations outside of codons 330–370 inactivate SMAD4 through the degradation of the protein (56). These data are pointing out the important role *SMAD4* is playing in HNSCC carcinogenesis (35). In this study, p.P296T and p.A488V mutant constructs did not abolish the rescuing effects of *SMAD4* overexpression on the migration and invasion in HNSCC cells, suggesting that these mutations may have other functions, warranting further research.

The loss of SMAD4 protein contributes to an increase in genomic instability in the tumor epithelia. This effect, together with blocking the growth inhibition and apoptosis which normally are induced by TGF-β but enhancing of TGF-β-mediated inflammation, could give way to the expansion of genetic defects cells during HNSCC tumorigenesis (57). *SMAD4* expression may be a determinant of sensitivity/resistance to EGFR/MAPK or EGFR/JNK inhibition in HPV-negative HNSCC tumors (58). However, the studies on SMAD4 loss have reported highly inconsistent results (30, 59, 60). Hernandez et al. developed a *SMAD4* fluorescence *in situ* hybridization assay to measure chromosomal *SMAD4* loss at the single-cell level in primary HNSCC samples and in and patient-derived xenografted (PDX) HNSCC tumors (61). They found a heterozygous loss of *SMAD4* in 35% of primary HNSCCs and 41.3% of PDX tumors. Moreover, in 4.3% of the PDX tumors, the loss of *SMAD4* was homozygous. Hernandez et al. also revealed intertumor and intratumor heterogeneities of *SMAD4* chromosomal loss in HNSCCs (60, 61). In the study presented here, LCM was used to purify the cancerous tissue because LCM makes it possible to approximate the true gene profile of pure cancer cell subpopulations in the context of their actual tissue environment (3, 4). Combining LCM and NGS may be used to detect changes in the karyotype of neoplastic lesions of the oral epithelium.

Analyzing a region of chromosome 18q which has been found to be frequently lost in pancreatic cancers led to the identification of *SMAD4* and the elucidation of its role in tumorigenesis (44), which was supported by the observation that germ-line *SMAD4* mutations cause juvenile polyposis (JP), a condition which is characterized by the formation of intestinal polyps at young age and a cumulative lifetime risk for gastrointestinal cancer of 50% (62). Breast cancers with mutations in the *SMAD4* gene (63) are as sensitive to PARP inhibitors as *BRCA*-mutant breast or ovarian cancers. Similar challenges exist for lung cancers with *SMAD4* (64), but a phenotype where *SMAD4* has been lost has not yet been observed.

A convenient tool to confirm the oncogenic effects of mutated genes in question is the controlled expression of mutant constructs. The current study revealed that mutant *SMAD4* can promote HNSCC tumorigenesis via cell migration and invasion. These data are concordant with the results of our clinical analysis, demonstrating more aggressive behavior and the potential for nodal metastasis of tumors with *SMAD4* mutations. The mechanisms that cause diverse *SMAD4* mutations, particularly missense mutations, confer loss-of-function to *SMAD4*.

In HNSCC, the region on chromosome 18q where *SMAD4* is located is frequently lost at the genetic level in HNSCC (65). In esophageal cancer, the loss of SMAD4 correlates with the invasion depth and the pathologic stage (59) as well as with regional metastases and with decreased survival (30).In animal models for HNSCC, *SMAD4* haploid insufficiency promoted tumor development (57). The loss of *SMAD4* is contributing to increased genomic instability in the tumor epithelia. Defects in the signaling of *SMAD* family proteins are associated with an increased tendency for metastatic spread and regional or distant recurrence of HNSCC (66). Thus, inactivation of TGF-β/SMAD signaling is frequently observed in HNSCC, and the inactivation of these signaling pathways might adversely affect patient outcomes. However, the location at which *SMAD4* is downregulated in human HNSCCs and the causal role of *SMAD4* LOH HNSCC development and progression remain unknown. We are the first to demonstrate that LOH and lower expression of SMAD4 can cause regional nodal metastasis and reduce the OS and DFS of HNSCC.

The inactivation of SMAD4 signaling is also associated with poorer prognosis in patients with adenocarcinoma of the pancreas and cancers of the esophagus (23, 43, 67). When *SMAD4* expression is lost in colorectal cancers (CRCs), it is associated with advanced stage disease, the presence of lymph node metastasis, and poor prognosis (38, 67, 68). However, Kouvidou et al. failed to illustrate this relationship in colon cancer (69). Bacman et al. observed that missing nuclear expression of *SMAD4* does not correlate with tumor grade or with the clinical outcome in colon cancer (70). Similar to CRCs, poor HNSCC-related patient outcomes are associated with 18q LOH (71, 72). SMAD4 depletion in an HNSCC cell line induces cetuximab resistance and results in worse survival in an orthotopic mouse model *in vivo*. JNK and MAPK activation as mediators of cetuximab resistance and provide the foundation for the concomitant EGFR and JNK/MAPK inhibition as a potential strategy for overcoming cetuximab resistance in HNSCCs with SMAD4 loss (58). However, in other studies, 18q loss did not appear to affect the survival of patients with HNSCCs (73, 74). This study indicates that LOH with *SMAD4* mutations may significantly decrease the survival of patients with HNSCCs.

Our analysis results demonstrated an inverse correlation between somatic *SMAD4* mutations and the downregulation of *SMAD4* in HNSCCs, following other studies. Furthermore, the results of our studies demonstrate that *SMAD4* loss due to mutations and downregulation results in increased tumor progression and recurrence rates. Moreover, *SMAD4* mutation and loss, as well as low *SMAD4* expression, worsened the OS. These factors may have led to the substantial differences between our and TCGA database's mutation profiles. Although the disparities warrant further resolution, the presented findings are congruent with those reported for other malignancies (75–77).

To summarize, HNSCC is highly heterogeneous at both the cellular and the genetic levels. The current findings show clearly that *SMAD4* expression is suppressing the progression of HNSCC. Somatic mutations in *SMAD4* and its expression determine the recurrence of HNSCC and go along with poor prognosis. Therefore, the proposed analysis of the genetic status may facilitate the identification of mutations in the *SMAD4* gene as a novel diagnostic marker or therapeutic target in HNSCC and other head and neck cancers.

## Data Availability Statement

The data in this manuscript was submitted to Short Reads Archive under the BioProject accession PRJNA588804 and SRA Run Selector project (https://www.ncbi.nlm.nih.gov/Traces/study/?acc=PRJNA588804&o=acc_s%3Aa).

## Ethics Statement

The studies involving human participants were reviewed and approved by MacKay Memorial Hospital. The patients/participants provided their written informed consent to participate in this study.

## Author Contributions

C-JL: study design. C-JL, L-HL, and K-WC: data analysis. H-WC and L-HL: lab work. C-JL and K-WC: paper preparation.

### Conflict of Interest

The authors declare that the research was conducted in the absence of any commercial or financial relationships that could be construed as a potential conflict of interest.

## Supplementary Material

Supplementary Figure 1 Identification of *SMAD4* mutations in HNSCC. **(A)** Schematic of five amplicons (blue arrows) covering exons 2–12 of *SMAD4* for sequencing. **(B)** Representative fluorescent electropherograms for LOH marker D18S363 (left), D18S474 (middle), and D18S46 (right). Samples of genomic DNA that were extracted from tumors and matching normal tissues were used as templates. **(C)** Representative Sanger sequencing analysis of mutations p.His132Tyr, p.Pro296Thr, and p.Ala488Val. Upper panels, blood samples; lower panels, cancer samples. The arrows depict wild-type or mutated nucleotides.

Supplementary Figure 2 Effect of si-*SMAD4* transfection in HNSCC cells. **(A)** Western blotting for OC4 (left) and OECM1 (right) treated with 20 and 40-nM si-*SMAD4* or control oligonucleotides for 48 h: **(B)** proliferation, **(C)** migration, and **(D)** invasion.

Supplementary Table 1 Clinicopathological characteristics of the HNSCC patients (*n* = 122).

Supplementary Table 2 List of long PCR primers used for amplification of *SMAD4* gene.

Supplementary Table 3 List of PCR primers used for PCR, sequencing, and construction, site directed mutagenesis, and *SMAD4* LOH.

Supplementary Table 4 List of *SMAD4* mutations in HNSCC tumors.

Supplementary Table 5 Clinical parameters and *SMAD4* LOH and/or mutations in HNSCC patients (*n* = 122).

Supplementary Table 6 Clinical parameter and *SMAD4* expression in OSCC patients (*n* = 70).

Supplementary Table 7 List of *SMAD4* mutations in HNSCC cell lines.
