# Supplementary figures and images for: SMAD4 Somatic Mutations in Head and Neck Carcinoma Are Associated With Tumor Progression
Source: Front Oncol. 2019 Dec 6;9:1379. doi: 10.3389/fonc.2019.01379 (PMC6909744; doi:10.3389/fonc.2019.01379)

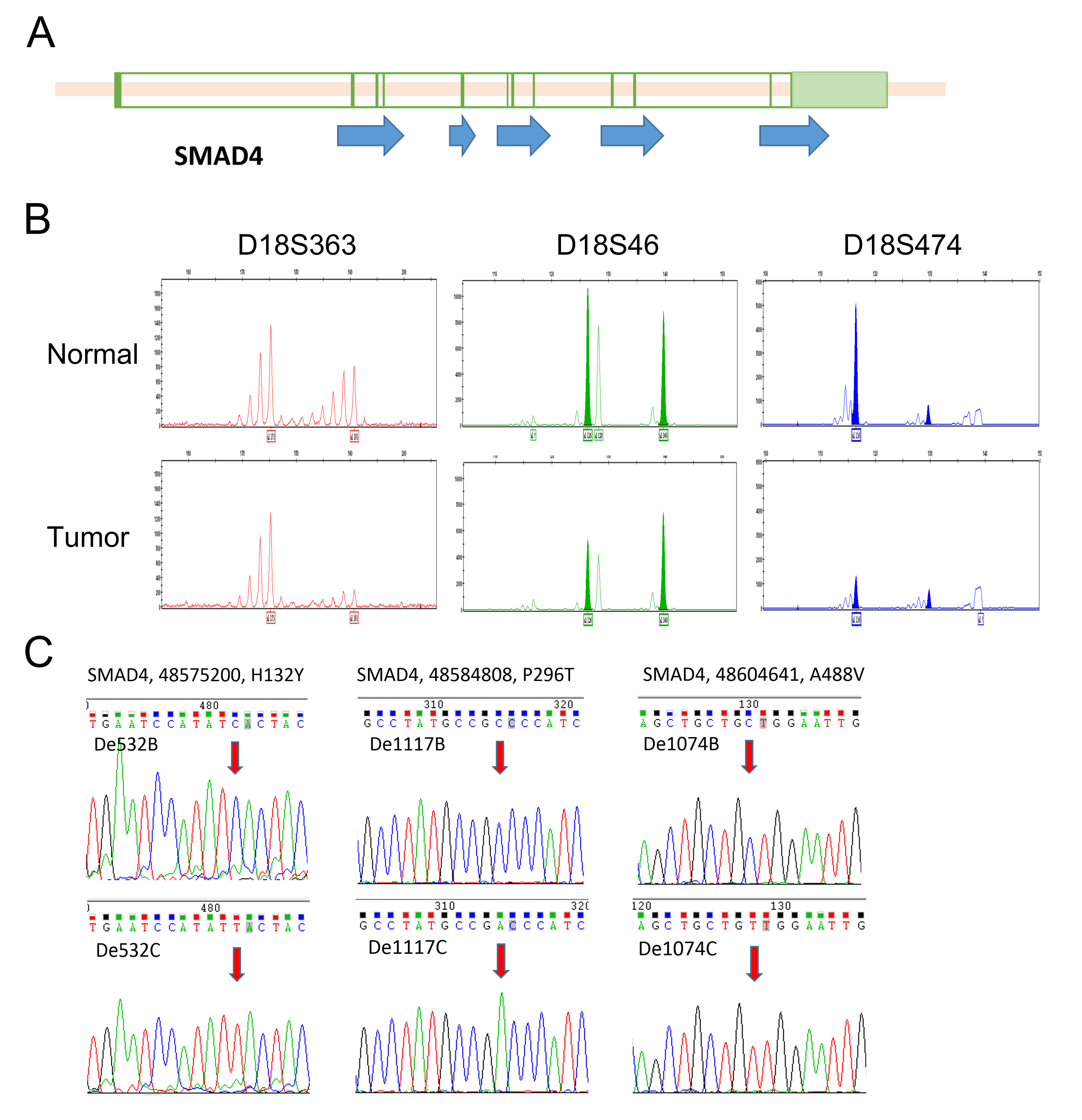

Supplement: Supplementary Figure 1 — Identification of SMAD4 mutations in HNSCC. (A) Schematic of five amplicons (blue arrows) covering exons 2–12 of SMAD4 for sequencing. (B) Representative fluorescent electropherograms for LOH marker D18S363 (left), D18S474 (middle), and D18S46 (right). Samples of genomic DNA that were extracted from tumors and matching normal tissues were used as templates. (C) Representative Sanger sequencing analysis of mutations p.His132Tyr, p.Pro296Thr, and p.Ala488Val. Upper panels, blood samples; lower panels, cancer samples. The arrows depict wild-type or mutated nucleotides. [file Image_1.TIF]

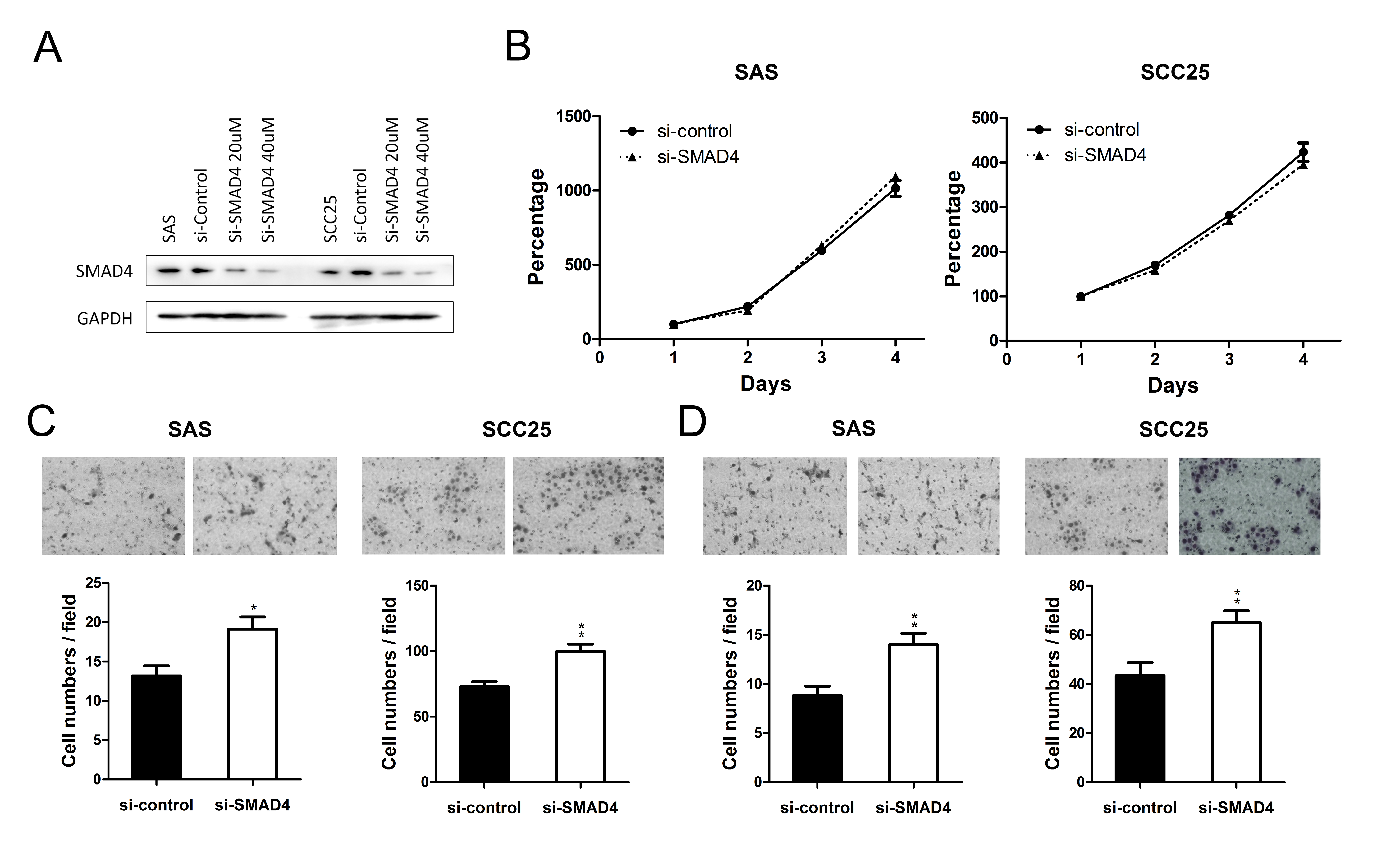

Supplement: Supplementary Figure 2 — Effect of si-SMAD4 transfection in HNSCC cells. (A) Western blotting for OC4 (left) and OECM1 (right) treated with 20 and 40-nM si-SMAD4 or control oligonucleotides for 48 h: (B) proliferation, (C) migration, and (D) invasion. [file Image_2.TIF]
