# Supplementary material for: SMAD4 Somatic Mutations in Head and Neck Carcinoma Are Associated With Tumor Progression
Source: Front Oncol. 2019 Dec 6;9:1379. doi: 10.3389/fonc.2019.01379 (PMC6909744; doi:10.3389/fonc.2019.01379)
Supplement: Supplementary Table 1 — Clinicopathological characteristics of the HNSCC patients (n = 122). [file Table_1.docx]

| **Supplementary Table 1.** Clinicopathological characteristics of the HNSCC patients (*n* = 122) | | |
| --- | --- | --- |
| Characteristics | HNSCC | |
| Follow-up (months) | 53.78 ± 3.53 | |
| Age (years) | 57.16 ± 0.98 | |
| Gender |  |  |
| Male | 112 | (91.8%) |
| Female | 10 | (8.2%) |
| Histological grade |  |  |
| Well | 49 | (40.2%) |
| Moderate | 64 | (52.5%) |
| Poor | 9 | (7.4%) |
| Clinical stage |  |  |
| I | 8 | (6.6%) |
| II | 12 | (9.8%) |
| III | 15 | (12.3%) |
| IV | 87 | (71.3%) |
| Tumor size |  |  |
| T1 | 10 | (8.2%) |
| T2 | 21 | (17.2%) |
| T3 | 12 | (9.8%) |
| T4 | 79 | (64.8%) |
| N stage |  |  |
| N0 | 73 | (59.8%) |
| N1 | 16 | (13.1%) |
| N2 | 33 | (27.1%) |
| Perineural invasion |  |  |
| No | 80 | (65.6%) |
| Yes | 42 | (34.4%) |
| Lymphovascular invasion |  |  |
| No | 94 | (77.0%) |
| Yes | 28 | (23.0%) |
