# Supplementary material for: SMAD4 Somatic Mutations in Head and Neck Carcinoma Are Associated With Tumor Progression
Source: Front Oncol. 2019 Dec 6;9:1379. doi: 10.3389/fonc.2019.01379 (PMC6909744; doi:10.3389/fonc.2019.01379)
Supplement: Supplementary Table 2 — List of long PCR primers used for amplification of SMAD4 gene. [file Table_2.docx]

| **Supplementary Table 2.** List of long PCR primers used for amplification of *SMAD4* gene | | | | |
| --- | --- | --- | --- | --- |
| Target | TM °C | Length (bp) | Primer ID | Sequence |
| EXON2-4 | 63.5 | 5712 | SMAD4-LRE2-4-forward | AAGGAAAGAGGCACGTCAGA |
|  |  |  | SMAD4-LRE2-4-reverse | AGGCAGTGAGTCTTCCTCCA |
|  |  |  |  |  |
| EXON5 | 60 | 3631 | SMAD4-LRE5-7-forward | TTTTGTGCCCCAAACAATTT |
|  |  |  | SMAD4-LRE5-a-reverse | CCCCCAGATTCTGCAAGTTA |
|  |  |  |  |  |
| EXON6-8 | 58 | 5885 | SMAD4-LRE6-8-forward | TGGAATAAATGGGGGACTGA |
|  |  |  | SMAD4-LRE6-8-reverse | GGGCGCTGTGTTTATGTTTT |
|  |  |  |  |  |
| EXON9-10 | 67 | 5120 | SMAD4-LRE9-10-forward | TGCACCTCTGTGAGAACAGG |
|  |  |  | SMAD4-LRE9-10-reverse | GCCACATAGGGTGAAGCAAT |
|  |  |  |  |  |
| EXON11-12 | 59 | 5761 | SMAD4-LRE11-12-forward | CTACGGGGGAGGCTAAAAAC |
|  |  |  | SMAD4-LRE11-12-reverse | CTGCACTGTTCACAGGAGGA |
