# Supplementary material for: SMAD4 Somatic Mutations in Head and Neck Carcinoma Are Associated With Tumor Progression
Source: Front Oncol. 2019 Dec 6;9:1379. doi: 10.3389/fonc.2019.01379 (PMC6909744; doi:10.3389/fonc.2019.01379)
Supplement: Supplementary Table 3 — List of PCR primers used for PCR, sequencing, and construction, site directed mutagenesis, and SMAD4 LOH. [file Table_3.docx]

| **Supplementary Table 3.** List of PCR primers used for PCR, sequencing, and construction, site directed mutagenesis, and *SMAD4* LOH. | | | | |
| --- | --- | --- | --- | --- |
| Target | Tm (℃) | Length (bp) | Primer ID | Sequence |
| **PCR primers and sequencing** | | | | |
| SMAD4 exon 3 | 55 | 375 | H132Y_Forward | TGAGTTGGTAGGATTGTGAGGA |
|  |  |  | H132Y _Reverse | TAAAGTCGCGGGCTATCTTC |
| SMAD4 exon 7 | 55 | 376 | P296T_Forward | AGGACAGCAGCAGAATGGAT |
|  |  |  | P296T_Reverse | TGAAACAAAATCACAGGATGAA |
|  |  |  | For sequence Reverse | AGCCCTTACAACAAAAACAAGA |
| SMAD4 exon 12 | 62 | 545 | A488V_Forward | AACCAAAAGTGTGCAGCTTG |
|  |  |  | A488V_Reverse | CTAGGAGCAAGGCAGCAAAC |
|  |  |  |  |  |
| **Construction** | | | | |
| SMAD4 cDNA | 55 | 1814 | SMAD4_Forward | CAA**GGATCC**TTGCAACGTTAGCTGTTG |
|  |  |  | SMAD4_Reverse | TTT**GAATTC**CACCATCCTGATAAGGTT |
|  |  |  |  |  |
| **Site directed mutagenesis** | | | | |
| SMAD4 cDNA | 55 | NA | H132Ymut | GTCTGTGTGAATCCATAT**T**ACTACGAACGAGTTG |
| SMAD4 cDNA | 55 | NA | P296Tmut | CTATGCCG**A**CCCATCCCGGACATTACTG |
| SMAD4 cDNA | 55 | NA | A488Vmut | GTCTGTCAGCTGCTG**T**TGGAATTGGTGTTGATG |
|  |  |  |  |  |
| ***SMAD4* LOH primer** | | | | |
| D18S363 | 60 | 177-247 | D18S363_Forward | (ROX) TTGGGAACTGCTCTACATTC |
|  |  |  | D18S363_Reverse | GCTTCATTCTCTCACTGGAT |
| D18S46 | 60 | 129-153 | D18S46_Forward | (HEX) GAATAGCAGGACCTATCAAAGAGC |
|  |  |  | D18S46_Reverse | CAGATTAAGTGAAAACAGCATATGTG |
| D18S474 | 60 | 119-139 | D18S474_Forward | (FAM) TGGGGTGTTTACCAGCATC |
|  |  |  | D18S474_Reverse | TGGCTTTCAATGTCAGAAGG |
