# Supplementary material for: SMAD4 Somatic Mutations in Head and Neck Carcinoma Are Associated With Tumor Progression
Source: Front Oncol. 2019 Dec 6;9:1379. doi: 10.3389/fonc.2019.01379 (PMC6909744; doi:10.3389/fonc.2019.01379)
Supplement: Supplementary Table 4 — List of SMAD4 mutations in HNSCC tumors. [file Table_4.docx]

| **Supplementary Table 4.** List of *SMAD4* mutations in HNSCC tumors | | | | | | |
| --- | --- | --- | --- | --- | --- | --- |
| Sample | Position | Ref | Alt | Classification | Coding change | Protein change |
| 1 | 48575200 | C | T | Missense | c.394C<T | p.His132Tyr |
| 1 | 48584808 | C | A | Missense | c.886C<A | p.Pro296Thr |
| 2 | 48573573 | G | A | Missense | c.157G<T | p.Glu53Lys |
| 3 | 48603052 | G | A | Synonymous | c.1353G<A | p.Ala451Ala |
| 4 | 48573586 | T | C | Missense | c.170C<A | p.Leu57Ser |
| 5 | 48573659 | G | A | Synonymous | c.243G<A | p.Arg81Arg |
| 5 | 48604641 | C | T | Missense | c.1463C<T | p.Ala488Val |
