# Supplementary material for: SMAD4 Somatic Mutations in Head and Neck Carcinoma Are Associated With Tumor Progression
Source: Front Oncol. 2019 Dec 6;9:1379. doi: 10.3389/fonc.2019.01379 (PMC6909744; doi:10.3389/fonc.2019.01379)
Supplement: Supplementary Table 5 — Clinical parameters and SMAD4 LOH and/or mutations in HNSCC patients (n = 122). [file Table_5.docx]

| **Supplementary Table 5.** Clinical parameters and *SMAD4* LOH and/or mutations in HNSCC patients (*n* = 122) | | | | | | | |
| --- | --- | --- | --- | --- | --- | --- | --- |
|  |  | *SMAD4* LOH and/or mutations | | | | |  |
| Characteristics | No. | No | | Yes | | | *P* value |
| Clinical stage |  |  |  | |  |  |  |
| I - II | 20 | 18 | (90.0%) | | 2 | (10.0%) | 0.097 |
| III - IV | 102 | 74 | (72.5%) | | 28 | (27.5%) |  |
| Tumor size |  |  |  | |  |  |  |
| T1 - T2 | 31 | 24 | (77.4%) | | 7 | (22.6%) | 0.764 |
| T3 - T4 | 91 | 68 | (74.7%) | | 23 | (25.3%) |  |
| N stage |  |  |  | |  |  |  |
| N0 | 73 | 61 | (83.6%) | | 12 | (16.4%) | 0.011* |
| N+ | 49 | 31 | (63.3%) | | 18 | (36.7%) |  |
| Perineural invasion |  |  |  | |  |  |  |
| No | 80 | 63 | (78.8%) | | 17 | (21.2%) | 0.237 |
| Yes | 42 | 29 | (69.0%) | | 13 | (31.0%) |  |
| Lymphovascular permeation | |  |  | |  |  |  |
| No | 94 | 71 | (75.5%) | | 23 | (245%) | 0.954 |
| Yes | 28 | 21 | (75.0%) | | 7 | (25.0%) |  |
| *P*-value calculated by chi-square test  *Statistically significant (*P*<0.05) | | |  | |  |  |  |
