# Supplementary material for: SMAD4 Somatic Mutations in Head and Neck Carcinoma Are Associated With Tumor Progression
Source: Front Oncol. 2019 Dec 6;9:1379. doi: 10.3389/fonc.2019.01379 (PMC6909744; doi:10.3389/fonc.2019.01379)
Supplement: Supplementary Table 6 — Clinical parameter and SMAD4 expression in OSCC patients (n = 70). [file Table_6.docx]

| **Supplementary Table 6.** Clinical parameter and SMAD4 expression in OSCC patients (n = 70) | | | | | | | | |
| --- | --- | --- | --- | --- | --- | --- | --- | --- |
|  |  |  | SMAD4 | | | |  |  |
| Status |  | No. | Low | | High | |  | *P* value |
| Clinical stage |  | |  |  |  |  |  |  |
| I - II |  | 12 | 6 | (50.0%) | 6 | (50.0%) |  | 0.004* |
| III - IV |  | 58 | 50 | (86.2%) | 8 | (13.8%) |  |  |
| Tumor size |  |  |  |  |  |  |  |  |
| T1 - T2 |  | 18 | 11 | (61.1%) | 7 | (38.9%) |  | 0.020* |
| T3 - T4 |  | 52 | 45 | (86.5%) | 7 | (13.5%) |  |  |
| N stage |  |  |  |  |  |  |  |  |
| N0 |  | 38 | 27 | (71.1%) | 11 | (28.9%) |  | 0.041* |
| N+ |  | 32 | 29 | (90.6%) | 3 | (9.4%) |  |  |
| Perineural invasion | | |  |  |  |  |  |  |
| No |  | 41 | 32 | (78.0%) | 9 | (22.0%) |  | 0.627 |
| Yes |  | 29 | 24 | (82.8%) | 5 | (17.2%) |  |  |
| Lymphovascular permeation | | | |  |  |  |  |  |
| No |  | 52 | 40 | (76.9%) | 12 | (23.1%) |  | 0.274 |
| Yes |  | 18 | 16 | (88.9%) | 2 | (11.1%) |  |  |
| *SMAD4* LOH and mutation | | |  |  |  |  |  |  |
| Wild type |  | 50 | 39 | (78.0%) | 11 | (22.0%) |  | 0.508 |
| LOH and/or mutations |  | 20 | 17 | (85.0%) | 3 | (15.0%) |  |  |
| *P*-value calculated by chi-square test  *Statistically significant (*P*<0.05) | | | | | | | | |
