# Supplementary material for: SMAD4 Somatic Mutations in Head and Neck Carcinoma Are Associated With Tumor Progression
Source: Front Oncol. 2019 Dec 6;9:1379. doi: 10.3389/fonc.2019.01379 (PMC6909744; doi:10.3389/fonc.2019.01379)
Supplement: Supplementary Table 7 — List of SMAD4 mutations in HNSCC cell lines. [file Table_7.docx]

| **Supplementary Table 7.** List of *SMAD4* mutations in HNSCC cell lines | | | | | | | | |
| --- | --- | --- | --- | --- | --- | --- | --- | --- |
|  |  | HNSCC cell line | | | | | | |
| Protein change | | OC3 | OC4 | OECM1 | HSC3 | SAS | SCC25 | Fadu |
| p.Lys106Glu | | v |  |  |  |  |  | NA |
| p.His132Tyr | |  |  |  | v |  |  | NA |
